# Children’s dental fear and anxiety: exploring family related factors

**DOI:** 10.1186/s12903-018-0553-z

**Published:** 2018-06-04

**Authors:** Lingli Wu, Xiaoli Gao

**Affiliations:** 1 Department of Dentistry, Key Laboratory of Oral Diseases of Gansu Province, Northwest University for Nationalities, Lanzhou, China; 2 Dental Public Health, Faculty of Dentistry, The University of Hong Kong, 3rd Floor, Prince Philip Dental Hospital, 34 Hospital Road, Sai Ying Pun, Hong Kong

**Keywords:** Dental fear, Dental anxiety, Children, Parents, Parenting styles, Family factors

## Abstract

**Background:**

Dental fear and anxiety (DFA) is a major issue affecting children’s oral health and clinical management. This study investigates the association between children’s DFA and family related factors, including parents’ DFA, parenting styles, family structure (nuclear or single-parent family), and presence of siblings.

**Methods:**

A total of 405 children (9–13 years old) and their parents were recruited from 3 elementary schools in Hong Kong. Child’s demographic and family-related information was collected through a questionnaire. Parents’ and child’s DFA were measured by using the Corah Dental Anxiety Scale (CDAS) and Children Fear Survey Schedule-Dental Subscale (CFSS–DS), respectively. Parenting styles were gauged by using the Parent Authority Questionnaire (PAQ).

**Results:**

DFA was reported by 33.1% of children. The mean (SD) CFSS-DS score was 29.1 (11.0). Children with siblings tended to report DFA (37.0% vs. 24.1%; *p* = 0.034) and had a higher CFSS-DS score (29.9 vs. 27.4; *p* = 0.025) as compared with their counterpart. Children from single-parent families had lower CFSS-DS score as compared with children from nuclear families (β = − 9.177; *p* = 0.029). Subgroup analysis showed a higher CFSS-DS score among boys with siblings (β = 7.130; *p* = 0.010) as compared with their counterpart; girls’ from single-parent families had a lower CFSS-DS score (β = − 13.933; *p* = 0.015) as compared with girls from nuclear families. Children’s DFA was not associated with parents’ DFA or parenting styles (*p* > 0.05).

**Conclusions:**

Family structure (nuclear or single-parent family) and presence of siblings are significant determinants for children’s DFA. Parental DFA and parenting style do not affect children’s DFA significantly.

**Electronic supplementary material:**

The online version of this article (10.1186/s12903-018-0553-z) contains supplementary material, which is available to authorized users.

## Background

Dental fear and dental anxiety (DFA) refer to the strong negative feelings associated with dental treatment, whether or not the criteria for a diagnosis of dental phobia are met [1]. The reported prevalence of DFA among children and adolescents in different countries ranged from 5 to 33% [1–4]. Children with DFA often try all means to avoid or delay dental treatment, resulting in deterioration of their oral health [5]. They also demonstrate poor cooperation during dental visits, which compromises the treatment outcomes, creates occupational stress on dental staff, and causes discord between dental professionals and their parents [1]. DFA acquisition in childhood may track into adulthood and is a significant predictor for dental avoidance in adulthood [6, 7]. Preventing and intercepting DFA during childhood is considered as a critical approach for improving people’s oral health and dental experience [5].

It was speculated that parents’ DFA might exerts an influence on their children’s DFA through modeling and information [8]. Many adults with DFA may verbalize their fearful feelings in front of their children, creating a negative impression on dental treatment [5]. Most children at early school age begin to emulate their parents who are looked upon as models [9]. They are very likely to internalize their parents’ values, attitudes and worldviews, which would gradually become a part of their own belief system [9]. There is moderate evidence to support the relationship between parental and child DFA [10]. An American study reported that over 40% of parents/guardians gave their children negative connotation about their previous dental visit [11]. This study also showed a shared anxiety between parents/guardians and their children, thus suggesting that parents played a key role in children’s anxiety and fear development. In another study, parental dental anxiety was demonstrated as a significant indicator for children’s dental anxiety (β = 0.244; *p* = 0.016) [12]. Despite the potential influence of parents, consensus is lacking regarding whether mother or father plays a more significant role in children’s DFA. A previous study concluded that fathers deliver major information, such as danger, to children and play a mediating role for the transfer of dental fear from parents to children [13]. However, another study showed no significant difference between the influence of mothers and fathers on their children’s DFA [14].

Parenting styles provided an environmental framework for children’s psychosocial growth and were assumed to shape children’s behaviors [15]. Baumrind identified three main styles of parenting, namely authoritative, authoritarian, and permissive [16]. This classification of parenting styles has served as a useful tool for investigating the influence of parenting on various issues concerning child development [16]. The relationship between parenting styles and children’s dental fear has attracted some scholarly attention. A study measured parents’ child-rearing attitudes and found that the subscale self-complaints (example item “My child’s happiness needs a lot of sacrifice on my part”) were associated with children’s dental fear [17]. However, another study found no association between parenting styles and dental anxiety of children [18]. With limited evidence gleaned from very few studies, the association between parenting styles and children’s DFA remains ambiguous.

In addition, a family dynamics model revealed that birth order could influence one’s personality and behavior [19]. It has been reported that children’s birth order partially determines their ability to cope with stresses in medical situations [20]. In the dental setting, only born children and first-born children were found to have a higher clinical situational DFA and were less cooperative than others [21, 22]. A study involving children of various age showed that more children reported to have DFA if their siblings reported DFA [23]. However, no association was found between children’s DFA and the number of children in their families in another study [24]. The current evidence concerning how birth order and presence of siblings are associated with children’s DFA remains scarce and inconsistent findings have been reported.

In view of the currently insufficient and contradictory evidence, this study aimed to investigate the association between children’s DFA and a variety of family related factors, namely parents’ DFA, parenting styles, family structure (single-parent or nuclear family), and presence of siblings.

## Methods

### Sample size calculation

The sample size was calculated by using G*Power version 3.1.9.2. Targeting a statistical power of 0.9 and a significant level of 0.05 and estimating 13.5% children have DFA [25], 373 subjects are needed to detect an effect size of 0.5.

### Participant recruitment

The protocol of this study was reviewed by the Institutional Review Board (IRB) of the University of Hong Kong/Hospital Authority Hong Kong West Cluster. An ethical approval was obtained (#UW16–130).

A list of government-funded elementary schools was retrieved from the official website of the Education Bureau, Hong Kong Special Administrative Region (http://www.edb.gov.hk). Among a total of 454 schools, 5 were randomly selected and approached. Three out of the 5 elementary schools participated. Child-mother-father triads were recruited. The inclusion criteria were: (i) the school was a government-funded, co-educational school (i.e. mixed-sex school); (ii) the child was enrolled in Primary 4–6 of a participating school; (iii) the child was 9–13 years old; and (iv) the child and his/her both parents were literate and were able to complete questionnaires themselves. Children with severe systemic diseases or physical or psychological disabilities were excluded. All eligible children in the participating schools were approached. Children with informed written consent from both parents were recruited.

### Questionnaires

Each participating family was asked to complete a set of four questionnaires. The questionnaires were distributed via class teachers and were completed by the participants at home on a self-administered basis. Clear instructions were given to avoid confusion. All questionnaires were completed anonymously. Each participating family was identified with a code and their names were not disclosed. The questionnaires were pretested among 6 families with diverse background to ensure relevance and clarity. Completing the questionnaires took approximately 20 min.

The first questionnaire, completed by parents, collected information on the child’s demographic background (age, gender, and birth place), family socioeconomic status (family income, parents’ education levels, parents’ occupation, and housing condition), family structure (single-parent or nuclear family), presence of sibling, and the birth order of the child.

The second questionnaire was the Parental Authority Questionnaire (PAQ); a psychometric scale that assesses the authoritativeness, authoritarianism and permissiveness practiced by fathers and mothers, respectively, in rearing their children. This study used a short version comprising 20 items, which was adapted from the Buri’s 30-item PAQ and showed adequate validity and internal consistency in children [26]. Test-retest reliability (the intraclass correlation) of Buri’s PAQ were 0.77–0.92 and internal consistency (Cronbach alpha) were 0.74–0.87 [27]. The validity was adequate; authoritarianism was inversely related to permissiveness (mother: *r* = − 0.38; father: *r* = − 0.50; all *p* < 0.0005) and authoritativeness (mother: *r* = − 0.48; father: *r* = − 0.52; all p < 0.0005) and permissiveness was not significantly related to authoritativeness (mother: *r* = 0.07; father: *r* = 0.12; all *p* > 0.10) [27]. The Chinese version of PAQ was adopted [28]. There were 7 items for authoritativeness, 7 for authoritarianism, and 6 for permissiveness. Responses to each item are made on a 5-point Likert scale, ranging from “strongly disagree” to “strongly agree”. The total score for each parenting style was calculated by summing scores of items in the corresponding parenting style. Among the three parenting styles (authoritative, authoritarian and permissive), the one with the highest mean score was regarded as the dominant parenting style for that parent. The PAQ was completed by the child him/herself, as instructed.

The third questionnaire was Corah Dental Anxiety Scale (CDAS) for measuring parents’ DFA. CDAS has been widely used in research studies and its reliability, validity and usefulness have been documented [29]. The test-retest reliability (correlation coefficient) was 0.82 and the internal consistency (Kuder-Richardson Formula) was 0.86. The validity was moderate as shown by the correlations between the dentists’ ratings and the patients’ test scores. The scale was later translated into Chinese [30]. CDAS contains 4 items, where respondents choose a score closest to their respective dental situations. The score ranged from 1 (not anxious) to 5 (extremely anxious). The total score for 4 items ranges from 4 to 20, with a higher score indicating a higher DFA level. The anxiety level was classified as “low” (scores below 9), “moderate” (scores from 9 to 12), “high” (scores from 13 to 14), and “severe” (scores from 15 to 20) [29].

The fourth questionnaire was the Children Fear Survey Schedule Dental Subscale (CFSS-DS), which was used to assess child’s DFA. CFSS-DS consists of 15 items on various anxiety stimuli [31]. To each item, the response ranges 1–5, from “not afraid at all” to “very much afraid”. The total score ranges from 15 to 75. Children with a total CFSS-DS score below 32 are considered non-fearful; 32–39 is defined as moderate fearful; > 39 is defined as fearful [31]. CFSS-DS is deemed one of the most commonly used psychological scales for children. A Chinese version of CFSS-DS [32] was used in this study and was completed by the child him/herself. The test-retest reliability (intraclass correlation) of CFSS-DS was 0.71 and the internal consistency (Cronbach’s alpha) was 0.85 [33]. The validity was good; higher mean CFSS-DS scores were found in children who were defined as uncooperative by using the Frankl Scale (standardized mean difference = 1.15; *p* < 0.01) [33]. All scales used can be found in Additional file 1.

### Statistical analysis

The data analysis was performed by using Statistical Package for Social Sciences (SPSS) version 23.0. Participants’ socio-demographic profile and family related factors were described. Parametric and non-parametric tests were used for comparing means/medians, whereas Chi-square tests were used for comparing proportions. Multiple linear regression models were constructed to test the associations after controlling for possible confounders. The collinearity among independent variables has been tested. In order to avoid possible collinearity, “mother’s education” and “father’s education” were converted to “parental education”, defined as education level of mother or father, whichever is lower. The same conversion applied to “parental occupation”. After such conversions, collinearity was ruled out because all the values of tolerance were well above 0.2. The stratified analysis by gender was also carried out to test the associations in boys and girls, respectively.

## Results

### Socio-demographic profile, family related factors and parenting styles

Among 881 eligible families approached, 405 participated and returned the questionnaires. The response rate was 46.0%. Most (71.0%) children were 10–11 year-old, with some 9 year-olds and 12–13 year-olds (Table 1). There were 188 (46.7%) boys and 215 (53.3%) girls. Most (90.3%) of them were born in Hong Kong. Around two-thirds (62.7%) of mothers were in the age group of 35–44 and two-thirds (60.7%) of fathers were 45 or above. Two-thirds (69.5%) of families had a moderate monthly income (HKD 10,000–39,999). Housing condition was classified into basic (tenement building, public permanent housing), moderate (home ownership scheme, village house, and dormitory) and good (owned or rent private housing estates). Around 61.5% lived in basic housing condition. The majority of mothers (74.3%) and fathers (72.8%) reported secondary school as their highest education level. Occupation is classified into managerial or professional (managers and administrators, professionals, self-employed), clerical or skilled workers (associate professionals, clerical support workers), service or labours (sales, unskilled laborers, service industry) and housewives/unemployed. Two-thirds (64.2%) of mothers were housewives. Two-thirds (40.8%) of fathers were in the industry of “service or labours”.

**Table 1:** Socio-demographic profiles of participants

		n	%
Child’s demographic			
Gender	Male	188	46.7
	Female	215	53.3
Age (years)	9	69	17.9
	10–11	274	71.0
	12–13	43	11.1
Place of birth	Hong Kong	355	90.3
	Other places	38	9.7
Parents’ demographic			
Mother’s age (years)	34 or below	45	12.1
	35–44	234	62.7
	45 or above	94	25.2
Father’s age (years)	34 or below	7	2.0
	35–44	131	37.3
	45 or above	213	60.7
Socio-economic status of the family			
Family monthly income^a^	Low (<HKD10000)	71	20.4
	Moderate (HKD10000–39999)	242	69.5
	High (≥HKD40000)	35	10.1
Housing condition^b^	Basic	236	61.5
	Moderate	46	12.0
	Good	102	26.6
Mother’s Education	Elementary school or below	33	8.7
	Secondary school	281	74.3
	Tertiary education or above	64	16.9
Father’s Education	Elementary school or below	39	11.0
	Secondary school	257	72.8
	Tertiary education	57	16.1
Mother’s Occupation^c^	Managerial or professional	25	6.4
	Clerical or skilled workers	51	13.0
	Service or labours	64	16.4
	Housewives	251	64.2
Father’s Occupation^c^	Managerial or professional	56	20.2
	Clerical or skilled workers	97	35.0
	Service or labours	113	40.8
	Unemployed	11	4.0
Total		405	100.0

^a^Source: Wang LD-L, Lam WWT, Fielding R. 2016. Hong Kong Chinese parental attitudes towards vaccination and associated socio-demographic disparities. Vaccine. 34:1426–1429

^b^Housing condition was classified into basic (tenement building, public permanent housing), moderate (home ownership scheme, village house, and dormitory) and good (owned or rent private housing estates)

^c^Occupation is classified into managerial or professional (managers and administrators, professionals, self-employed), clerical or skilled workers (associate professionals, clerical support workers), service or labours (sales, unskilled laborers, service industry) and housewives/unemployed

The majority (91.5%) of children were from nuclear families. Most (70.3%) children had siblings and more than half (55.9%) were the first child in the family. Among the three parenting styles, both parents scored the highest in authoritativeness. The means (SD) were 3.6 (0.8) and 3.6 (0.9) for mothers and for fathers respectively. This was followed by “authoritarian”, for which the means (SD) were 3.1 (0.8) and 3.0 (0.9) for mothers and fathers respectively. The scores were the lowest in permissiveness, with a mean (SD) of 2.6 (0.8) and 2.8 (0.9) for mothers and fathers respectively. “Authoritative” was identified as the dominant parenting style for 68.6 and 72.1% mothers and fathers respectively; while 25.0% mothers and 18.0% fathers practiced authoritarian parenting. Only 6.4% mothers and 9.9% fathers were permissive.

### Dental fear and anxiety (DFA) of child and parents

Table 2 shows children’s responses to possible fearful events related to dental practice. Items that over 20% children felt “very much afraid” or “pretty much afraid” were “the dentist drilling” (32.1%), “the sight of dentist drilling” (24.3%), “having a stranger touch you” (23.6%), “hearing drilling sound” (22.5%) and “injection” (20.6%). As for their DFA level, 66.9% of the children were considered non-fearful, 15.3% were moderate and 17.8% were fearful. The mean (SD) total CFSS-DS score was 29.1 (11.0).

**Table 2:** Children’s dental fear and anxiety (DFA)

	Not afraid at all	Very little fear	Moderate sfear	Pretty much afraid	Very much afraid
Events/possible triggers	n (%)				
Dentists	187 (47.3)	147 (37.2)	27 (6.8)	12 (3.0)	22 (5.6)
Doctors	276 (69.9)	83 (21.0)	16 (4.1)	9 (2.3)	11 (2.8)
Injections	122 (31.0)	139 (35.3)	52 (13.2)	26 (6.6)	55 (14.0)
Somebody examines your mouth	265 (67.3)	93 (23.6)	24 (6.1)	6 (1.5)	6 (1.5)
Having to open your mouth	334 (84.8)	44 (11.2)	7 (1.8)	3 (0.8)	6 (1.5)
Having a stranger touch you	100 (25.3)	120 (30.4)	82 (20.8)	37 (9.4)	56 (14.2)
Having somebody look at you	180 (45.8)	107 (27.2)	61 (15.5)	22 (5.6)	23 (5.9)
Dentist drilling	97 (24.6)	92 (23.3)	79 (20.0)	42 (10.6)	85 (21.5)
Sight of dentist drilling	168 (43.0)	76 (19.4)	52 (13.3)	36 (9.2)	59 (15.1)
Hearing drilling sound	155 (39.6)	92 (23.5)	56 (14.3)	30 (7.7)	58 (14.8)
Putting instruments in mouth	172 (43.8)	102 (26.0)	46 (11.7)	25 (6.4)	48 (12.2)
Choking	163 (42.0)	116 (29.9)	58 (14.9)	22 (5.7)	29 (7.5)
Having to go to the hospital	185 (47.4)	103 (26.4)	53 (13.6)	18 (4.6)	31 (7.9)
People in white uniform	302 (77.0)	56 (14.3)	15 (3.8)	8 (2.0)	11 (2.8)
Having the dentist clean your teeth	271 (69.0)	77 (19.6)	19 (4.8)	11 (2.8)	15 (3.8)
Mean (SD) of total CFSS-DS score	29.1 (11.0)				
Range of total CFSS-DS score	15.0–66.0				
Possible range of the scale	15.0–75.0				
Level of DFA	Non-fearful CFSS-DS score < 32		Moderate fear CFSS-DS score 32–39	Fearful CFSS-DS score > 39	
	n (%)				
	245 (66.9)		56 (15.3)	65 (17.8)	

The DFA level of 43.4, 42.1, 11.6 and 2.8% mothers was considered low, moderate, high and severe respectively. As for fathers’ DFA level, 54.7, 37.0, 6.6 and 1.7% were considered low, moderate, high and severe respectively. The means (SD) total CDAS score of mothers and fathers were 9.2 (3.0) and 8.2 (2.9) respectively.

### Family related factors and children’s DFA

No significant difference was found in children’s DFA among all socio-economic subgroups (all *p* > 0.05). Table 3 shows the results of bivariate analysis between each family related factor and children’s DFA. Parental DFA and parenting styles of both parents were not associated with children’s DFA (all p > 0.05). Children having siblings tended to report DFA, as compared with single child (37.0% vs. 24.1%; *p* = 0.034). They also had a higher CFSS-DS score (29.9 vs. 27.4; *p* = 0.025). No significant difference was found in DFA scores of those who were first child in the family and others.

**Table 3:** Family related factors and Children’s DFA

	No fear (CFSS-DS <32)	Moderate fear (CFSS-DS 32-39)	Severe fear (CFSS-DS >39)	Total CFSS-DS score
		n (%)		Mean (SD)
Parental dental fear				
CDAS scale of mother				
Low anxiety	109 (69.4)	27 (17.2)	21 (13.4)	27.90 (10.02)
Moderate anxiety	90 (63.8)	20 (14.2)	31 (22.0)	29.72 (11.98)
High/sever anxiety	35 (66.0)	8 (15.1)	10 (18.9)	30.49 (10.62)
		*P* = 0.411		*P* = 0.277
CDAS scale of father				
Low anxiety	128 (70.3)	21 (11.5)	33 (18.1)	28.42 (11.75)
Moderate anxiety	78 (62.4)	22 (17.6)	25 (20.0)	29.80 (10.63)
High/sever anxiety	13 (56.5)	7 (30.4)	3 (13.0)	31.39 (11.10)
		*P* = 0.141		*P* = 0.111
Parenting style				
Mother dominant style				
Authoritative	167 (68.2)	33 (13.5)	45 (18.4)	28.91 (10.75)
Authoritarian	58 (63.7)	18 (19.8)	15 (16.5)	29.54 (11.64)
Permissive	16 (69.6)	2 (8.7)	5 (21.7)	28.87 (13.23)
		*P* = 0.593		*P* = 0.853
Father dominant style				
Authoritative	164 (66.9)	39 (15.9)	42 (17.1)	28.56 (10.63)
Authoritarian	42 (64.6)	10 (15.4)	13 (20.0)	30.52 (11.74)
Permissive	24 (70.6)	4 (11.8)	6 (17.6)	29.54 (13.22)
		*P* = 0.952		*P* = 0.531
Family characteristics				
Family structure				
Nuclear family	212 (66.3)	51 (15.9)	57 (17.8)	29.1 (11.1)
Single-parent family	23 (76.7)	3 (10.0)	4 (13.3)	26.3 (9.6)
		*p* = 0.500		*p* = 0.167
Having siblings				
Yes	158 (62.9)	45 (17.9)	48 (19.1)	29.9 (11.1)
No	85 (75.9)	10 (8.9)	17 (15.2)	27.4 (10.8)
		p = 0.034*		p = 0.025*
First child				
Yes	141 (70.5)	22 (11.0)	37 (18.5)	28.8 (11.4)
No	98 (62.8)	31 (19.9)	27 (17.3)	29.4 (10.4)
		*p* = 0.065		*p* = 0.280

*p* values for categorical outcomes were obtained from the Chi-square test. *p* values for continuous outcomes were obtained from the non-parametric test (Mann-Whitney U test or Kruskal-Wallis test)

*Significant difference

When multivariate analysis (linear regression) was conducted (Table 4), children from single-parent families were found with lower CFSS-DS score as compared with children from nuclear families (β = − 9.177; *p* = 0.029). When stratified analysis was carried out for boys and girls separately (Model 2 and Model 3), it was found that (i) boys who had siblings had significantly higher DFA in contrast to those without siblings (β = 7.130; *p* = 0.010); (ii) boys whose family had a basic housing condition reported significantly lower DFA as compared with those under a good housing condition (β = − 7.752; *p* = 0.006); and (iii) girls from single-parent families had lower DFA score as compared with girls from nuclear families (β = − 13.933; *p* = 0.015).

**Table 4:** Determinants of DFA in children (CFSS-DS score)

		Model 1 (all children)	Model 2 (boys)	Model 3 (girls)
		β (95% CI)**	β (95% CI)**	β (95% CI)**
Age (years)	Continuous	−0.165 (−1.229, 0.899)	0.822 (− 1.339, 2.982)	− 0.838 (− 2.146, 0.470)
Gender	Male ^#^			
	Female	2.554 (−0.088, 5.197)	─	─
Place of birth	Hong Kong^#^			
	Other places	−4.036 (− 8.848,0.776)	0.839 (− 6.055, 7.733)	− 6.143 (− 13.355, 1.068)
Family monthly income	Continuous	−0.098 (−1.342, 1.146)	− 1.057 (− 2.835, 0.721)	0.964 (− 0.925, 2.854)
Family housing condition	Good^#^			
	Moderate	−3.373 (− 8.068, 1.322)	−7.011 (− 13.943, − 0.079)	−2.977(− 9.927, 3.972)
	Basic	−3.247 (− 6.857,0.363)	−7.752 (− 13.204, − 2.301)*	− 2.061 (− 7.240, 3.117)
Parental Education^##^	Continuous	−2.214 (− 5.415, 0.986)	−4.540 (− 9.825, 0.745)	−1.907 (− 6.314, 2.500)
Parental Occupation^##^	Unemployed/housewives^#^			
	Service /labours	3.223 (−0.014, 6.460)	5.646 (1.220, 10.071)*	1.309 (− 3.608, 6.225)
	Clerical/skilled workers	−0.434 (− 4.916, 4.048)	−5.057 (− 12.018, 1.904)	−0.509 (− 6.841, 5.822)
	Managerial/ professional	2.748 (− 6.035, 11.530)	15.713 (− 0.516, 31.942)	− 4.041 (− 15.161, 7.079)
Age of first dental visit	1–6 years-old ^#^			
	7–11 years-old	−0.153 (− 3.857, 3.551)	−3.001 (− 8.119, 2.117)	3.416 (− 2.348, 9.179)
First visit as checkup	Checkup ^#^			
	Treatment	−0.749 (− 3.844, 2.347)	1.520 (−3.098, 6.138)	− 1.885 (− 6.179, 2.409)
Having sibling	No ^#^			
	Yes	3.386 (−0.210, 6.982)	7.130 (1.757, 12.503)*	1.147 (− 4.228, 6.523)
First child	No ^#^			
	Yes	1.038 (−2.317, 4.394)	3.159 (−2.090, 8.408)	− 0.010 (− 4.761, 4.740)
Family structure	Nuclear family^#^			
	Single-parent family	−9.177(− 17.418,-0.936)*	− 4.564 (− 17.936, 8.808)	−13.933(− 25.136,-2.731)*
Mother’s dominant parenting style	Authoritative ^#^			
	Authoritarian	0.062 (−3.283, 3.407)	0.222 (−4.494, 4.938)	− 0.504 (− 5.392, 4.385)
	Permissive	2.277 (−4.071, 8.624)	0.651 (− 10.264, 11.566)	2.921 (−5.681, 11.523)
Father’s dominant parenting style	Authoritative ^#^			
	Authoritarian	0.099 (−3.662, 3.859)	0.720 (−4.730, 6.170)	0.383 (−5.239, 6.006)
	Permissive	1.689 (−2.723, 6.101)	4.298 (−1.834, 10.430)	−1.052 (− 7.703, 5.599)
Mother’s dental fear	Continuous	0.209 (−0.251, 0.669)	0.388 (−0.267, 1.042)	0.044 (− 0.627, 0.716)
Father’s dental fear	Continuous	0.298 (−0.178, 0.774)	0.310 (−0.344, 0.965)	0.310 (− 0.407, 1.027)
Constant		28.845(13.512, 44.177)	22.804(−5.129, 50.737)	37.964 (17.833,58.095)
		R^2^ = 0.095	R^2^ = 0.224	R^2^ = 0.117

The results were obtained through multiple linear regressions using total CFSS–DS score of all children, CFSS–DS score of boys, and CFSS–DS score of girls as a dependent variable respectively. Independent variables are as above

*Significant difference between/among subgroups

**CI: confidence interval

^#^Reference group

^##^Parental education was defined as mother’s or father’s education, whichever is lower. The same conversion applied to “parental occupation”

## Discussion

This study explored the possible associations between children’s DFA and a variety of family related factors. The results showed that DFA was quite common an issue reported by around one third (33.1%) of children. In comparison with children from nuclear families, children from single-parent families had lower DFA score. Subgroup analysis showed that boys’ DFA was associated with “having siblings”, whereas girls’ DFA was lower when they were from “single-parent families”. Children’s DFA was however not associated with parents’ DFA or parental styles.

Invasive procedures and dental pain were often reported as the most important causes of dental anxiety [5, 14]. Similarly, teenagers participating in this study tended to relate their DFA to injection and drilling; the latter also extended to stimuli (sound and sight) associated with drilling. It is worth noting that “having a stranger touch you” also appeared as a major fearful situation for the respondents. Our finding is somewhat in line with that of a previous study, in which “having a stranger touch you” was identified as the highest-ranked cause for dental fear, followed by “injection” and “choking” [34]. Although being touched by a stranger in the clinical scenario may not be unexpected and stressful to adult patients, it may impose considerable stress on adolescents. This highlights the importance of building rapport and trust with adolescent patient before starting any dental examination and treatment.

It was often speculated that parental DFA and parenting styles are associated with children’s DFA. Such association was supported by a studies conducted in the US [12] and was however absent in our study sample. It is worth noting that our study focused on a slightly older age group as compared with the American study. There is a notion that older children’s perception of dental treatment is more influenced by their actual dental experience such as painful procedure and professional’s behaviors [17]. Fear toward unknown appears to be predominant during early childhood, but fears are usually linked to body injures including various situations encounter in dental setting by 9 years of age [35]. A review also indicated that the relationship between parental and child’ dental fear was obvious in children under 8 years old [10]. It is possible that parental DFA is not a direct factor to influence child’s DFA when the child reached adolescence.

The finding that children in single-parent families reported less DFA is in congruence with a previous study [18] but contradict some others [36, 37]. In single-parent families, children may become more independent and are more likely to grow maturity and resilience [38]. Given these characteristics, they may cope better with the challenges and stressful situations, such as dental visit. Also, children in single-parent families may receive more attention from other caregivers, such as grandparents. This might also play a role in their growth and development. For instance, grandfathers were identified as an important figure who can affect the emotional transmission of dental fear among family members [13].

Given the gender difference in psychological development and socialization, stratified analysis was performed for boys and girls respectively. It was found that among boys, the impact of having sibling(s) was evident; those with sibling(s) reported significantly higher DFA. There is a notion that only-born children tend to internalize parents’ expectations, turn predominantly mature, have higher self-esteem, and grow adult behavior due to their enhanced time interacting with adults [39]. On the other hand, there was an alternative theory describing personality of single-born children as more self-centered, demanding, shy, dependent and moody [40]. While the former lends support to our findings, the latter points to the possibility of higher DFA in single children, which was a finding of some other studies [21, 22]. There were also research studies suggesting no difference in the personalities of single child and their peers with siblings when judged by parents, teachers, and themselves [41]_._ Apart from personality traits, siblings’ past dental experience and their positive or negative modeling might play an important role in shaping children’s perception of dental care. The analysis for girls showed that living in a single family indicated a lower level of DFA, as compared with those in nuclear families. It is believed that girls are more adult-oriented [42]; therefore they may be more affected by parental factors and less by their siblings.

The findings of this study could be better interpreted by taking into consideration the methodological strengthens and limitations. Data were collected from a relatively large sample with diverse backgrounds. Well-established and validated psychometric scales were used to measure parenting styles and the DFA of children and their parents. For determining the parenting styles, different scales are available and can be completed by parents or children. Since parents may tend to give socially desirable answers and their perceptions may not truly reflect the reality, children’s response is preferable. Although some items may seem abstract, reliable answers could be obtained from adolescents through proper wording of the questions. This study is cross-sectional in nature. Therefore, no temporal relationship can be established and our findings could only suggest associations but not causation. The sample of this study was drawn from children in Hong Kong. Therefore, findings of this study cannot be directly extrapolated to other populations, although some useful implications can be drawn especially for populations of similar cultures and social context. Although the regression models suggested the impact of familial factors on children’s DFA, the R^2^ were low and the models only explained 9.5, 22.4, and 11.7% of the variance in DFA of all subjects, boys, and girls, respectively. This supports the notion that DFA is a complex phenomenon, in which many other factors, such as child’s personality traits [43], past dental experiences [44], other life incidents/events [45], were involved.

To sum up, several implications can be derived from the findings of this study. The commonly neglected factors (e.g. “having a stranger touch you”) that trigger DFA in children implies that dental personnel could consider spending some time wherever possible to establish trust with paediatric patients before proceeding to dental procedures. Previous research revolved around parents’ DFA and consequently negative modeling on children. Our findings however suggest that the impact of family on the development of children’s DFA is not as straightforward as previously speculated. In contrast to the assumption held by many people, our results showed that children’s DFA is not associated with parents’ DFA and parenting style. Instead, family structure (nuclear/single-parent family) and presence of siblings plays a significant role in children’s DFA. To prevent and intercept DFA among children, it may be important to redirect the attention from parental influence to possible negative influence of siblings. Although children from nuclear family might benefit from such healthy family environment in many aspects of their personal growth, they are more likely to have DFA. Parents and clinicians are advised to be more sensitive to the signs of DFA in these children and make necessary efforts to prepare them for dental visits.

## Conclusions

Family structure (nuclear or single-parent family) and presence of siblings are significant determinants for children’s DFA. Parental DFA and parenting style do not affect children’s DFA significantly.

## Additional file

Additional file 1: Scales used (PDF 265 kb)

## Abbreviations

CDAS: Corah Dental Anxiety Scale
CFSS–DS: Fear Survey Schedule-Dental Subscale
DFA: Dental fear and anxiety
PAQ: Parent Authority Questionnaire
SPSS: Statistical Package for Social Sciences
