# Supplementary material for: Children’s dental fear and anxiety: exploring family related factors
Source: BMC Oral Health. 2018 Jun 4;18:100. doi: 10.1186/s12903-018-0553-z (PMC5987456; doi:10.1186/s12903-018-0553-z)
Supplement: Supplementary file 1 — Scales used (PDF 265 kb) [file 12903_2018_553_MOESM1_ESM.pdf]

## Parental Authority Questionnaire (PAQ)

**Instructions:** For each of the following statements, circle the number of the 5-point scale that best describes how that statement applies to you and your **MOTHER**.

1 = Strongly disagree 2 = Disagree 3 = Neutral 4 = Agree 5 = Strongly Agree

| | |
|---|---|
| (1) Once family rules had been established, my mother discussed the reasoning behind the rules with the children. | 1 2 3 4 5 |
| (2) My mother directed the activities and decisions of the children in the family through reasoning and discipline. | 1 2 3 4 5 |
| (3) As the children in my family were growing up, my mother consistently gave us direction and guidance in reasonable and objective ways. | 1 2 3 4 5 |
| (4) My mother had clear standards of behaviour for the children in our home, but she was willing to adjust those standards to the needs of each of the individual children in the family. | 1 2 3 4 5 |
| (5) My mother gave me direction for my behavior and activities as I was growing up and she expected me to follow her direction, but she was always willing to listen to my concerns and to discuss that direction with me. | 1 2 3 4 5 |
| (6) As I was growing up my mother gave me clear direction for my behaviors and activities, but she was also understanding when I disagreed with her. | 1 2 3 4 5 |
| (7) As I was growing up, if my mother made a decision in the family that hurt me, she was willing to discuss that decision with me and to admit it if she had made a mistake. | 1 2 3 4 5 |
| (8) Even if her children didn't agree with her, my mother felt that it was for our own good if we were forced to conform to what she thought was right. | 1 2 3 4 5 |
| (9) Whenever my mother told me to do something as I was growing up, she expected me to do it immediately without asking any questions. | 1 2 3 4 5 |
| (10) My mother has always felt that more force should be used by parents in order to get their children to behave the way they are supposed to. | 1 2 3 4 5 |
| (11) My mother felt that wise parents should teach their children early who is boss in the family. | 1 2 3 4 5 |
| (12) As I was growing up my mother would get very upset if I tried to disagree with her. | 1 2 3 4 5 |
| (13) As I was growing up my mother let me know what behavior she expected of me, and if I didn't meet those expectations, she punished me. | 1 2 3 4 5 |
| (14) My mother has always felt that most problems in society would be solved if parents strictly and forcibly dealt with their children when they don't do what they are supposed to. | 1 2 3 4 5 |
| (15) My mother has always felt that children need to be free to make up their own minds and to do what they want to do, even if this does not agree with what their parents might want. | 1 2 3 4 5 |
| (16) As I was growing up, my mother did not feel that I needed to obey rules and regulations of behavior simply because someone in authority has established them. | 1 2 3 4 5 |
| (17) As I was growing up, my mother seldom gave me expectations and guidelines for my behavior. | 1 2 3 4 5 |
| (18) My mother feels that most problems in society would be solved if parents did not restrict their children's activities, decisions, and desires. | 1 2 3 4 5 |
| (19) My mother did not view herself as responsible for directing and guiding my behavior as I was growing up. | 1 2 3 4 5 |
| (20) My mother did not direct the behaviors, activities, and desires of the children in the family. | 1 2 3 4 5 |

**Instructions:** For each of the following statements, circle the number of the 5-point scale that best describes how that statement applies to you and your **FATHER**.

1 = Strongly disagree  2 = Disagree  3 = Neutral  4 = Agree  5 = Strongly Agree

| | |
|---|---|
| (1) Once family rules had been established, my father discussed the reasoning behind the policy with the children. | 1 2 3 4 5 |
| (2) My father directed the activities and decisions of the children in the family through reasoning and discipline. | 1 2 3 4 5 |
| (3) As the children in my family were growing up, my father consistently gave us direction and guidance in reasonable and objective ways. | 1 2 3 4 5 |
| (4) My father had clear standards of behavior for the children in our home, but he was willing to adjust those standards to the needs of each of the individual children in the family. | 1 2 3 4 5 |
| (5) My father gave me direction for my behavior and activities as I was growing up and he expected me to follow his direction, but he was always willing to listen to my concerns and to discuss that direction with me. | 1 2 3 4 5 |
| (6) As I was growing up my father gave me clear direction for my behaviors and activities, but he was also understanding when I disagreed with him. | 1 2 3 4 5 |
| (7) As I was growing up, if my father made a decision in the family that hurt me, he was willing to discuss that decision with me and to admit it if he had made a mistake. | 1 2 3 4 5 |
| (8) Even if his children didn't agree with him, my father felt that it was for our own good if we were forced to conform to what he thought was right. | 1 2 3 4 5 |
| (9) Whenever my father told me to do something as I was growing up, he expected me to do it immediately without asking any questions. | 1 2 3 4 5 |
| (10) My father has always felt that more force should be used by parents in order to get their children to behave the way they are supposed to. | 1 2 3 4 5 |
| (11) My father felt that wise parents should teach their children early who is boss in the family. | 1 2 3 4 5 |
| (12) As I was growing up my father would get very upset if I tried to disagree with him. | 1 2 3 4 5 |
| (13) As I was growing up my father let me know what behavior he expected of me, and if I didn't meet those expectations, he punished me. | 1 2 3 4 5 |
| (14) My father has always felt that most problems in society would be solved if parents strictly and forcibly dealt with their children when they don't do what they are supposed to. | 1 2 3 4 5 |
| (15) My father has always felt that children need to be free to make up their own minds and to do what they want to do, even if this does not agree with what their parents might want. | 1 2 3 4 5 |
| (16) As I was growing up, my father did not feel that I needed to obey rules and regulations of behavior simply because someone in authority has established them. | 1 2 3 4 5 |
| (17) As I was growing up, my father seldom gave me expectations and guidelines for my behavior. | 1 2 3 4 5 |
| (18) My father feels that most problems in society would be solved if parents did not restrict their children's activities, decisions, and desires. | 1 2 3 4 5 |
| (19) My father did not view himself as responsible for directing and guiding my behavior as I was growing up. | 1 2 3 4 5 |
| (20) My father did not direct the behaviors, activities, and desires of the children in the family. | 1 2 3 4 5 |

## Corah Dental Anxiety Scale (CDAS)

If you had to go to the dentist tomorrow for a check-up, how would you feel about it?

a. I would look forward to it as a reasonably enjoyable experience.
b. I wouldn't care one way or the other.
c. I would be a little uneasy about it.
d. I would be afraid that it would be unpleasant and painful.
e. I would be very frightened of what the dentist would do.

When you are waiting in the dentist's office for your turn in the chair, how do you feel?

a. Relaxed
b. A little uneasy
c. Tense
d. Anxious
e. So anxious that I sometimes break out in a sweat or almost feel physically sick

When you are in the dentist's chair waiting while the dentist gets the drill ready to begin working on your teeth, how do you feel?

a. Relaxed
b. A little uneasy
c. Tense
d. Anxious
e. So anxious that I sometimes break out in a sweat or almost feel physically sick

Imagine you are in the dentist's chair to have your teeth cleaned. While you are waiting and the dentist or hygienist is getting out the instruments which will be used to scrape your teeth around the gums, how do you feel?

a. Relaxed
b. A little uneasy
c. Tense
d. Anxious
e. So anxious that I sometimes break out in a sweat or almost feel physically sick

## Children Fear Survey Schedule Dental Subscale (CFSS-DS)

| What do you feel to: | Not afraid at all | Very little fear | Moderate fear | Pretty much afraid | Very much afraid |
|---|---|---|---|---|---|
| Dentists | ☐ | ☐ | ☐ | ☐ | ☐ |
| Doctors | ☐ | ☐ | ☐ | ☐ | ☐ |
| Injections | ☐ | ☐ | ☐ | ☐ | ☐ |
| Having somebody examine your mouth | ☐ | ☐ | ☐ | ☐ | ☐ |
| Having to open your mouth | ☐ | ☐ | ☐ | ☐ | ☐ |
| Having a stranger touch you | ☐ | ☐ | ☐ | ☐ | ☐ |
| Having somebody look at you | ☐ | ☐ | ☐ | ☐ | ☐ |
| The dentist drilling | ☐ | ☐ | ☐ | ☐ | ☐ |
| The sight of dentist drilling | ☐ | ☐ | ☐ | ☐ | ☐ |
| The noise of the dentist drilling | ☐ | ☐ | ☐ | ☐ | ☐ |
| Having somebody put instruments in your mouth | ☐ | ☐ | ☐ | ☐ | ☐ |
| Choking | ☐ | ☐ | ☐ | ☐ | ☐ |
| Having to go to the hospital | ☐ | ☐ | ☐ | ☐ | ☐ |
| People in white uniform | ☐ | ☐ | ☐ | ☐ | ☐ |
| Having the dentist clean your teeth | ☐ | ☐ | ☐ | ☐ | ☐ |
